# 229 Evaluation of the Global Matrix of Para Report Cards on Physical Activity of Children and Adolescents with Disabilities

**DOI:** 10.1093/eurpub/ckae114.062

**Published:** 2024-09-26

**Authors:** Farid Bardid, Yeshayahu Hutzler, Salomé Aubert, Jurate Pozeriene, Kwok Ng

**Affiliations:** University of Strathclyde, Glasgow, United Kingdom; Levinsky-Wingate Academic College, Netanya, Israel; Active Healthy Kids Global Alliance, Ottawa, Canada; Lithuanian Sports University, Kaunas, Lithuania; Lithuanian Sports University, Kaunas, Lithuania; University of Limerick, Limerick, Ireland; University of Turku, Rauma, Finland

## Abstract

**Purpose:**

Physical activity provides a wide range of health benefits for children and adolescents with disabilities (CAWD). However, despite CAWD being at higher risk of physical inactivity, there is a lack of surveillance systems that capture physical activity data of CAWD. To address this gap, the Global Matrix of Para Report Cards on physical activity of CAWD was set up with 14 participating countries/jurisdictions (Ng et al., 2023) based on the methodology of the Active Healthy Kids Global Alliance’s Global Matrix 4.0 (Aubert et al., 2023). This study aims to evaluate the process, outcome, and impact of the Global Matrix of Para Report Cards.

**Methods:**

The evaluation was informed by the Global Matrix 3.0 evaluation process (Aubert et al., 2020). Process, outcome, and impact indicators were informed by online surveys, reports/publications, and website analytics of the special issue published in Adapted Physical Activity Quarterly.

**Results:**

Two online surveys have been completed by 64-71% of targeted respondents. Moderate-to-high satisfaction rates were reported for the development of and participation in the Global Matrix initiative and open-ended comments reported issues (e.g., data availability, inconsistency in definitions) and positive feedback (e.g., networking/collaboration, visibility). The participating Report Card teams assigned and contributed a total of 139 physical activity grades—overall physical activity, organised sport, active play, active transport, physical fitness, sedentary behaviour, family and peers, schools, community and environment, government—to the Global Matrix of which 45% were incomplete. Twelve Report Card teams published a short Para Report Card article for their country. As of February 2024, >47,000 views have been recorded for the special issue comprising the Global Matrix and country Para Report Card articles.

**Conclusions:**

This evaluation highlights the need for more discussion on the definitions of disability and benchmarks of physical activity indicators to improve the grading process and more support in accessing and synthesising data related to CAWD. These are important steps to support inclusion and representation of CAWD in (inter)national surveillance of physical activity and health.

